# MetaboMiner – semi-automated identification of metabolites from 2D NMR spectra of complex biofluids

**DOI:** 10.1186/1471-2105-9-507

**Published:** 2008-11-28

**Authors:** Jianguo Xia, Trent C Bjorndahl, Peter Tang, David S Wishart

**Affiliations:** 1 Department of Biological Sciences, University of Alberta, Edmonton, AB, T6G 2E9, Canada; 2 Department of Computing Science, University of Alberta, Edmonton, AB, T6G 2E8, Canada; 3 National Institute for Nanotechnology, 11421 Saskatchewan Drive, Edmonton, AB, T6G 2M9, Canada

## Abstract

**Background:**

One-dimensional (1D) ^1^H nuclear magnetic resonance (NMR) spectroscopy is widely used in metabolomic studies involving biofluids and tissue extracts. There are several software packages that support compound identification and quantification via 1D ^1^H NMR by spectral fitting techniques. Because 1D ^1^H NMR spectra are characterized by extensive peak overlap or spectral congestion, two-dimensional (2D) NMR, with its increased spectral resolution, could potentially improve and even automate compound identification or quantification. However, the lack of dedicated software for this purpose significantly restricts the application of 2D NMR methods to most metabolomic studies.

**Results:**

We describe a standalone graphics software tool, called MetaboMiner, which can be used to automatically or semi-automatically identify metabolites in complex biofluids from 2D NMR spectra. MetaboMiner is able to handle both ^1^H-^1^H total correlation spectroscopy (TOCSY) and ^1^H-^13^C heteronuclear single quantum correlation (HSQC) data. It identifies compounds by comparing 2D spectral patterns in the NMR spectrum of the biofluid mixture with specially constructed libraries containing reference spectra of ~500 pure compounds. Tests using a variety of synthetic and real spectra of compound mixtures showed that MetaboMiner is able to identify >80% of detectable metabolites from good quality NMR spectra.

**Conclusion:**

MetaboMiner is a freely available, easy-to-use, NMR-based metabolomics tool that facilitates automatic peak processing, rapid compound identification, and facile spectrum annotation from either 2D TOCSY or HSQC spectra. Using comprehensive reference libraries coupled with robust algorithms for peak matching and compound identification, the program greatly simplifies the process of metabolite identification in complex 2D NMR spectra.

## Background

Metabolomics is a rapidly-growing field of "-omics" research concerned with the high-throughput comparison, identification and quantification of large numbers of metabolites in biological systems [1]. Metabolomics only became possible as a result of recent technology breakthroughs in small molecule separation, identification and quantification. These include advances in ultra-sensitive, ultra-precise mass spectrometry (MS), robotic, multi-dimensional NMR spectroscopy, and greatly improved HPLC and UPLC separation technologies [2]. While other techniques may be more sensitive or less expensive, NMR has emerged as an ideal platform for studying metabolites in biofluids. It is a rapid, highly precise, non-destructive, and quantitative technique that allows one to compare, identify and quantify a wide range of compounds without the need for prior compound separation or derivatization [3-6]. NMR is particularly amenable to compounds that are less tractable to GC-MS or LC-MS analysis, such as sugars, amines, volatile ketones and relatively non-reactive compounds. A key disadvantage of NMR is that it is a relatively insensitive technique, with a lower limit of detection of 1~5 μM and a requirement of relatively large sample sizes (~500 μL).

Currently, most NMR-based metabolomic studies involve the analysis of 1D ^1^H NMR spectra, although 1D ^13^C and ^31^P NMR spectra may also be analyzed [7-10]. There are generally two routes to analyzing NMR spectra for metabolomic studies. In one method (called the chemometric approach), the compounds are not initially identified – only their spectral patterns and intensities are recorded and statistically compared in order to identify the relevant spectral features that distinguish sample classes. Once these features have been located, a variety of approaches may then be used to identify the corresponding metabolites [11]. In the other approach (often called quantitative metabolomics or targeted profiling), compounds are first identified and quantified by comparing the NMR spectrum of the biofluid of interest to a spectral reference library obtained from pure compounds [12]. Once these compounds are identified and quantified, the data can be analyzed in many different ways to identify the most relevant biomarkers or informative pathways.

A variety of protocols and software tools have been recently developed for conducting quantitative metabolomics via 1D ^1^H NMR [5,6,12,13]. In most cases, a manual peak-fitting process is required in order to perform compound identification and quantification. However, this manual fitting process can become particularly difficult and prone to frequent errors, especially for very complex biofluid mixtures (such as urine or tissue extracts) due to severe spectral overlap. In contrast to 1D NMR, 2D NMR offers a robust approach to resolving excessively overlapped spectra. Indeed 2D (and 3D) NMR has long been used to resolve and identify individual resonances from large macromolecules such as DNA, RNA and proteins. 2D NMR experiments such as TOCSY, HSQC and J-resolved spectroscopy are also increasingly being used in metabolomic studies in order to resolve spectral ambiguities to aid in the identification of specific compounds in complex biofluid mixtures [14-22].

A number of small-molecule NMR databases have been developed in recent years to support metabolomics research, including the Human Metabolome Database (HMDB) [23], the BioMagResBank Database (BMRB) [24], the Madison Metabolomics Consortium Database (MMCD) [25], the Magnetic Resonance Metabolomics Database (MRMD) [26], and the Platform for RIKEN Metabolomics (PRIMe). These resources, which contain significant numbers of reference NMR spectra of metabolites, also support metabolite identification through web-based submission of 1D and 2D NMR peak lists. However, these on-line tools don't generally provide graphical support for peak filtering, processing, comparative display or annotation. Furthermore, they don't exploit additional constraints such as knowledge about biofluid composition or metabolite concentration ranges to make compound identification even more robust, more accurate, and more efficient. We propose that by using a comprehensive 2D spectral reference library along with a more detailed knowledge of metabolite compositions of different biofluids, it is possible to greatly improve compound identification from 2D NMR spectra. Here we report our efforts in developing an easy-to-use, graphically-based, stand-alone software tool, called MetaboMiner, to perform semi-automated metabolite identification from 2D TOCSY and HSQC-spectra of complex biofluid mixtures.

## Methods

### Data Collection and Curation

Key to the development of this software package was the creation of an extensive 2D spectral library containing TOCSY and HSQC spectra of pure metabolites. We used several publicly available sources in constructing this library. The majority of the raw ^1^H-^1^H TOCSY spectra were collected from the standard compound spectral library available at the BMRB database [24]. A few additional compound spectra were obtained from the MRMD [26]. The ^1^H-^13^C HSQC spectral library was downloaded from the HMDB [23]. These raw spectra contained a number of spectral artefacts (noise, water bands, asymmetries, peaks from TSP or DSS, contaminants, *etc*.). Consequently it was necessary to convert these raw spectra into "synthetic" or "simplified" spectra corresponding to the peaks specific to the pure compounds of interest. This conversion was done manually, with each of these simplified, noise-free spectra being examined for inconsistencies by comparing them to the original raw spectra and the compound's known resonance assignments. In total, the MetaboMiner TOCSY reference library includes spectra from 223 common metabolites and the MetaboMiner HSQC library contains spectra from 502 metabolites. The compounds in both libraries were further catalogued into three sub-libraries corresponding to the three common human biofluids – cerebrospinal fluid (CSF), plasma and urine. The classification was based on their respective metabolic compositions listed in the HMDB [23]. Since the presence of these biofluid-specific metabolites was determined by a variety of technologies not limited to NMR, we further investigated the appearance of these metabolites in a large number of 1D ^1^H spectra collected from CSF, plasma and urine. The combined collection of compounds (and spectra) were used to create corresponding "common biofluid" 2D NMR spectral libraries that effectively represent a generic biofluid or cell extract. The "CSF", "plasma", "urine", "biofluid" and "total" spectral libraries are stored as XML files and are editable via MetaboMiner's graphical user interface (GUI).

**Figure 1:**
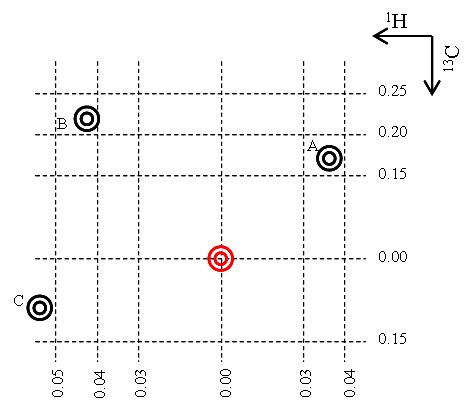
**An illustration of the calculation of uniqueness values**. The red peak represents the peak of interest and three peaks are in its immediate vicinity. The calculations are only performed at five chemical shifts distance levels – 0.01, 0.02, 0.03, 0.04, 0.05 ppm along the ^1^H dimension, and 0.05, 0.10, 0.15, 0.20, 0.25 ppm along the ^13^C dimension. No peak is observed in the first three distance levels. So the maximum unique scope for this peak is (0.03, 0.15) ppm. Peak A is found within 0.03~0.04 ppm (^1^H dimension) and 0.15~0.20 ppm (^13^C dimension) of the red peak; Peak B is found within 0.04~0.05 ppm (^1^H dimension) and 0.20~0.25 ppm (^13^C dimension) of the red peak; Peak C is not considered since the chemical shift distance is more than 0.05 ppm along the ^1^H dimension. Therefore, the assigned uniqueness values are 0-0-0-1-2. Note that the distance is not drawn to scale.

After the spectral libraries were constructed, each peak for each compound in each library was assigned a series of uniqueness values that are specific for that reference library. A unique peak in MetaboMiner is defined as a relatively isolated peak around which no peak from any other compound is observed based on the spectral library of the given biofluid. For any given peak, its uniqueness value is calculated as the total number of surrounding peaks from other compounds within a given chemical shift "distance". Five distance levels were used to measure peak uniqueness. For ^1^H chemical shifts, the distance thresholds are 0.01, 0.02, 0.03, 0.04, and 0.05 ppm. For ^13^C chemical shifts, the distance thresholds are set at 0.05, 0.10, 0.15, 0.20, and 0.25 ppm. For instance, an HSQC peak with a series of assigned uniqueness values of 0-0-0-1-2 indicates that no peak from any other compound in the reference library is observed within 0.03 ppm (^1^H dimension) and 0.15 ppm (^13^C dimension) of that peak. It also indicates that one peak from another compound in the spectra library was observed within 0.03~0.04 ppm (^1^H dimension) and 0.15~0.20 ppm (^13^C dimension) and another peak from another compound was observed within 0.04~0.05 ppm (^1^H dimension) and 0.20~0.25 ppm (^13^C dimension). See Figure 1 for a more complete description of the uniqueness value concept. These uniqueness values are automatically updated after any spectral library change using MetaboMiner's graphical user interface.

### Peak Processing, Peak Matching and Compound Identification

As part of its input, MetaboMiner requires peak lists corresponding to the peaks that were identified in either the TOCSY or HSQC spectra collected from the biofluid(s) of interest. While it is possible for users to provide manually picked peak lists, MetaboMiner also supports processing of multidimensional NMR peak lists obtained from automatic peak peaking programs. Multidimensional NMR spectra typically contain substantial numbers of spectral artefacts such as baseline distortions, intense solvent lines, ridges, sinc wiggles, *etc*. Automatic peak-picking programs tend to mistake these noise signals for real resonances. Therefore, any raw 2D spectra collected from biofluids must be processed appropriately before attempting to match them to MetaboMiner's reference spectral library. Two automated procedures were found to be very effective in cleaning up raw 2D spectra: 1) streak removal and 2) symmetrical editing. Note that the latter processing technique is only applicable for TOCSY spectra. Spectral streaks are usually caused by residual solvent signals (i.e. water) or the presence of other compounds at extremely high concentrations. Streaks can be recognized by their specific locations and prominent shapes in the NMR spectra. Streak removal was implemented by searching for groups of peaks at these common locations and eliminating them from the peak list. Symmetrical editing exploits the fact that real TOCSY peak signals form a symmetrical square pattern along the diagonal line. Off-diagonal peaks without any corresponding symmetrical peaks can be considered to be artefacts. Both peak positions and intensities (if provided by the user) of the corresponding peaks are examined for symmetry. Since TOCSY cross peaks are frequently not of equal intensity we require that the intensity ratio between the upper and lower-diagonal peaks should be within a range of 0.8~2.5 of each other to be considered symmetrical.

In order to accommodate small chemical shift differences between the observed NMR spectra and the reference NMR spectra, an adaptive threshold method was implemented based on the uniqueness values (described above) of each reference peak. During the peak searching/matching process, the search threshold varies automatically based on the maximum uniqueness value of the current peak. For instance, when searching for potential matches for a TOCSY peak with uniqueness values of 0-0-0-0-1, MetaboMiner will automatically set its threshold to 0.04 ppm. The peak matching and adaptive thresholding employ two processes: a reverse search strategy and a forward search strategy. In the reverse search strategy, the library peaks are searched and matched against the query peaks. Typically most query peaks find their potential matches during this reverse search step. However there are usually some peaks left without any matches. In order to assign these unmatched peaks a forward search is performed in which the unmatched query peaks are searched against the reference library with expanded thresholds – 0.08 ppm for TOCSY and 0.12 ppm (^1^H) and 0.4 ppm (^13^C) for HSQC spectra. A match is identified if only a single reference peak is identified within this range.

In MetaboMiner a compound is considered to be present only if its matched pattern satisfies the requirements of what we call "minimal signatures". A minimal signature is defined as the minimum peak set that can uniquely identify a compound from all others in a given spectral library. Based on the complete peak set of the reference spectral library, many minimal signatures can be derived through different combinations of unique peaks. A single peak match may be considered a minimal signature if it is completely unique. More peaks are required to define a minimal signature for less unique ones. For instance, in our current implementation, the presence of a single peak with uniqueness values 0-0-0-0-x (x >= 0) will determine the presence of the corresponding compound (subject to authenticity checks as discussed later); while at least two peaks with uniqueness values 0-0-0-x-x are required to reach the decision.

Since query spectra (i.e. real spectra from biofluids) usually contain substantial levels of spectral noise, even after pre-processing, we found that we could reduce MetaboMiner's false positive rate even further by implementing several authenticity checks. These include: 1) having a minimum number of matched peaks (3 for TOCSY spectra and 1 for HSQC spectra), 2) having a minimum matched fraction of peaks (1/2 for TOCSY spectra and 1/6 for HSQC spectra), 3) ensuring the presence of certain peaks for certain compounds (determined by manual testing and validation for each compound), and 4) ensuring that the identified compounds were known to be in a given biofluid.

### User Interface Description

**Figure 2:**
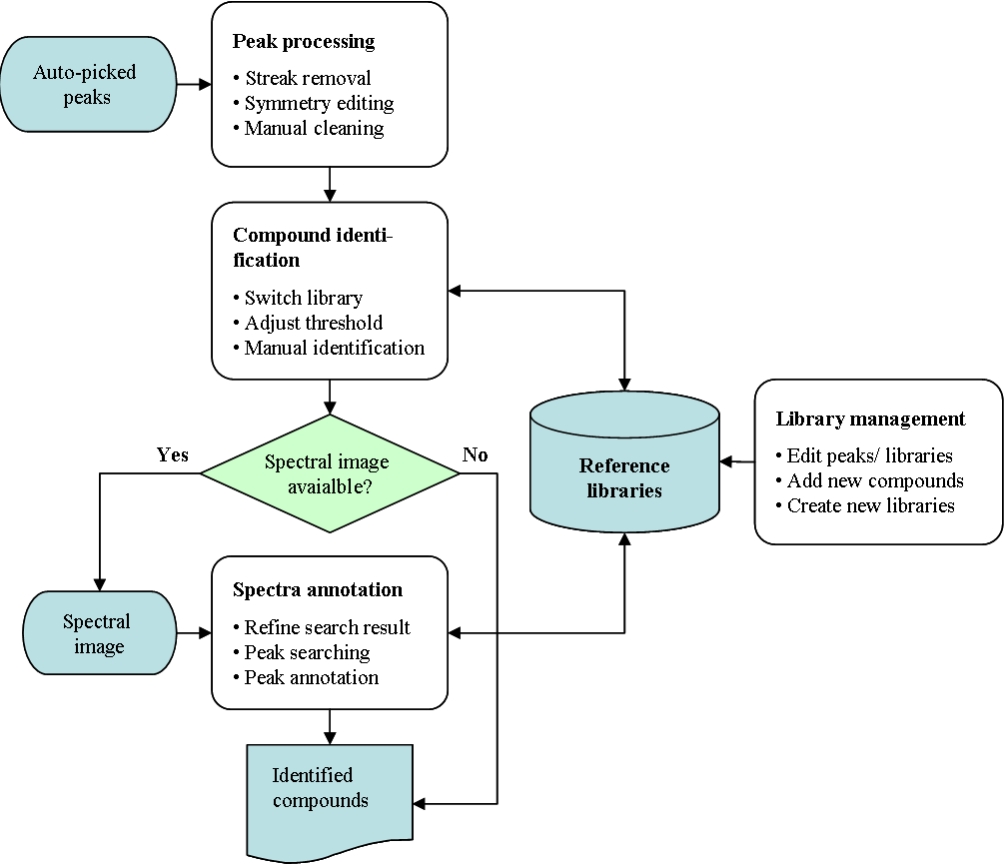
**MetaboMiner flowchart**. The query peaks obtained from an automatic peak-picking program are first processed to remove streaks and other artefacts. The cleaned peak list is then scanned for the presence of peak patterns of compounds in a spectral reference library corresponding to the biofluid that has been identified by the user. Spectral images can be used to further refine the search result.

MetaboMiner's graphical user interface was implemented using Java Swing technology. The spectral visualization and manipulation tools were built using the JGraph library (Java open source graph visualization library, ). Figure 2 illustrates a flowchart describing the MetaboMiner GUI. There are four main functional views, 1) a Processing View, 2) a Search View, 3) an Annotation View, and 4) a Library View. All these views share the same component arrangement, with panels on the right side being used for visualizing and manipulating peaks, and the panels on the left being used for displaying parameters, compound lists, structure images, *etc*. Navigation to each view is readily accessible by clicking an appropriate menu item.

**Figure 3:**
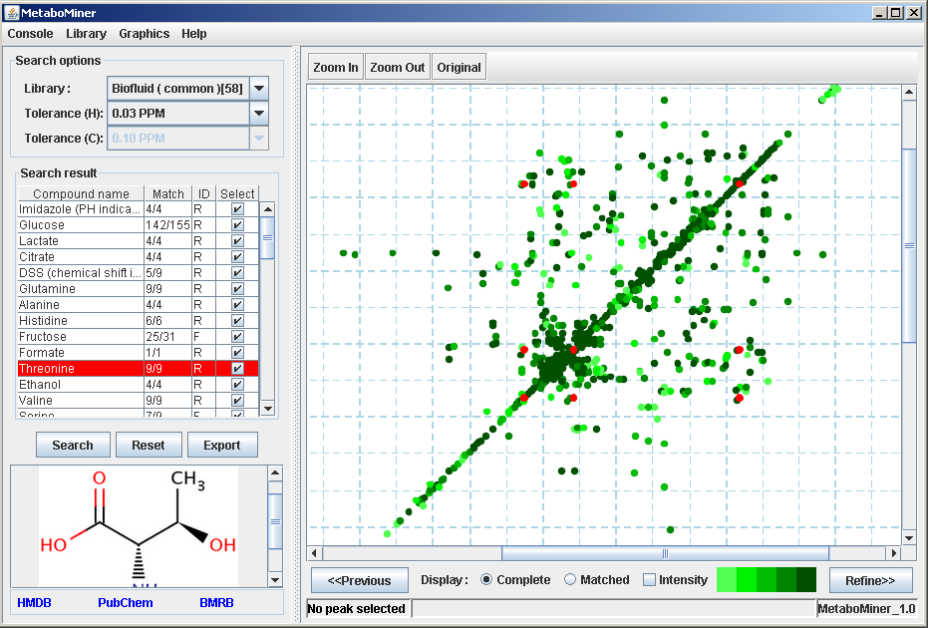
**Screenshot of MetaboMiner's "Search View"**. The left panel shows the library compounds that have matches in the query peaks. The selected checkbox indicates the corresponding compound is considered to be present by MetaboMiner. 'R' or 'F' indicates whether the compound is identified during the reverse search or forward search, respectively. On the right panel, the reference peaks (in red) of the current selected compound is displayed with query peaks as background. The color variations represent the peak intensities with the dark green corresponding to the strongest peak intensities. When the mouse is placed over any synthetic peak, all its information (name, position, uniqueness values, *etc*.) will be displayed on the view panel. Right clicking on any peak will allow users to search the spectral library for this particular peak.

When the program launches, the default view is the "Processing View" where users can copy and paste the automatically picked peak list. The input format must be either a two or three-column list, with numbers separated by a space or a semicolon. The first two columns must be the x and y chemical shift coordinates of each peak in the 2D spectrum and the optional third column must be the peak height or peak intensity. After processing the raw peaks, both the original and the processed spectra will be displayed on MetaboMiner's spectral viewing panel (located on the right). With this viewing panel, users can directly edit peaks on the spectrum if necessary. For manually picked peaks, this step can be skipped by turning the processing options off. By clicking the "Search" button, MetaboMiner's "Search View" will be displayed with its initial, automated compound identification results. Users can adjust the search threshold or switch the reference library to further refine the result. A compound is marked as identified if the matched pattern passes the authenticity checks and satisfies the minimum signature requirement. The raw matched scores are also displayed. MetaboMiner's interface allows users to visually inspect the matched peaks of any metabolite against the corresponding reference spectrum. By right clicking any peak displayed on the spectrum, users can search the library for this particular peak. The identified compound list can be saved in three different formats by clicking the "Export" button. A screenshot of MetaboMiner's "Search View" is shown in Figure 3.

**Figure 4:**
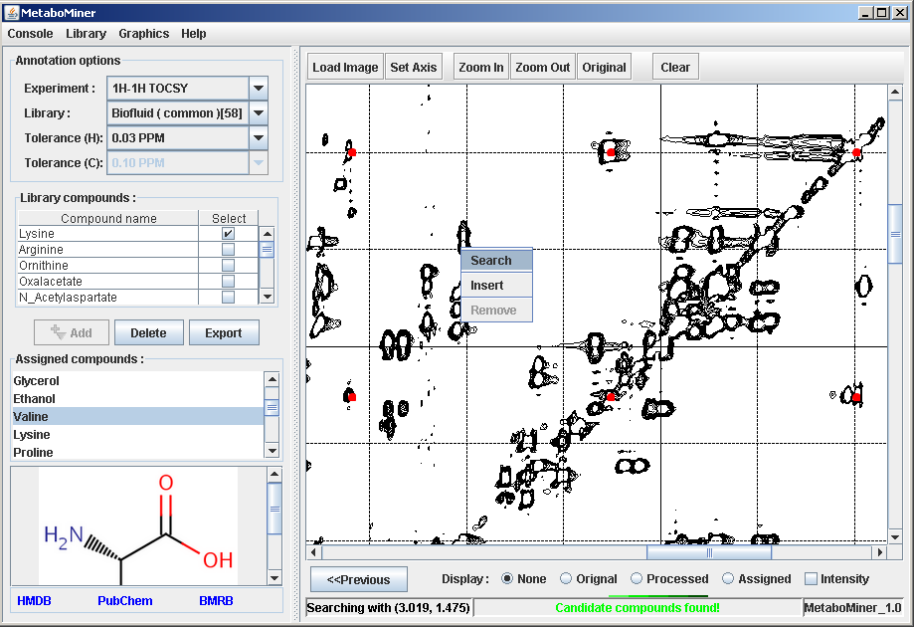
**Screenshot of MetaboMiner's "Annotation View"**. The contents of the reference spectral library and the automatically identified compound list are shown on the left panel. The spectral image is displayed on the right panel. The red peaks correspond to the reference spectra of the current compound being annotated (Valine). Peak searching is carried out by right clicking on a corresponding Valine peak. The user can also directly edit the current compound by inserting, removing, or dragging its peaks to match the exact pattern of the reference spectrum.

Users can further refine the automated search results by manually annotating the raw 2D spectrum. By clicking the "Refine" button in the "Search View", the "Annotation View" will be launched with the identified compounds being transferred as the starting point. Users can also directly enter the "Annotation View" mode by clicking the "Annotate" button from the "Console" menu. In order to perform manual annotation, users first need to load a high resolution spectral image in PNG format and set up the spectral axes properly. Peak searching is performed by right clicking the peak position on the spectrum to search the reference library as shown in Figure 4. All compounds that generate peaks within the search threshold will be checked. The compound with the closest peak match will be highlighted with its database reference spectrum displayed on the uploaded "raw" spectrum. Users can perform peak annotation for any currently displayed compound. Double clicking any database peak will open a small text editor where users can enter the peak assignment or a comment. The peak pattern of the identified compounds can also be edited to match the experimental spectrum. For example, users can insert, delete, or drag a database peak to match the observed peak in the raw spectrum. These changes will be valid only for the current session. To make permanent changes, MetaboMiner's "Library View" must be used.

The "Library View" is intended for browsing and managing MetaboMiner's spectral libraries. To view all the available reference spectra in MetaboMiner's libraries, users must click the "Browse" button in the "Library" menu. Double clicking any compound in the compound list will open a popup window for peak editing. Any changes will be reflected on the spectrum at real time. New compounds can be introduced by clicking the "New" button at the bottom of the compound list. A new compound can be either exported from another library or be created from scratch through the wizard dialog. Both peak editing or adding new compounds will trigger updating of the uniqueness values of the affected peaks. For researchers who study other types of biological samples (e.g. plant or microbial extracts), they may either use MetaboMiner's generic spectral reference library or create a new library customized for that particular type of biofluid. Library creation or deletion can be easily accomplished by clicking the appropriate menu items in the "Library" menu. The compounds in the default reference library are linked to PubChem, HMDB [18], and the BMRB [19] via the hyperlink under their structure icon. The "Graphics" menu enables users to change the size, shape, or color of the synthetic peaks to suit their preferences.

It is important to note that MetaboMiner does not support spectral processing such as phasing, baseline correction or chemical shift referencing. There are many other high-quality NMR-processing software available for this task, including NMRPipe [27], Felix (Molecular Simulations, Inc., San Diego, CA), VNMR (Varian, Inc., Palo Alto, CA), and XWinNMR (Bruker Analytik GmbH, Karlsruhe, Germany), to name a few. These tools should be used prior to loading spectral images into MetaboMiner. In other words, MetaboMiner is not a spectral processing tool, but a NMR-based metabolomics tool that facilitates automatic peak processing, rapid compound identification, and facile spectrum annotation capabilities through an intuitive graphical interface. MetaboMiner is available at: 

### Evaluation protocol

MetaboMiner was assessed in a variety of ways using both synthetic and experimental NMR spectra. The synthetic spectra were generated from the 162 compounds that have both TOCSY and HSQC spectra in the reference library. The experimental spectra were collected from three defined compound mixtures (totalling 72 compounds) and a biofluid sample of known composition (plasma). These evaluations allowed a complete and comprehensive assessment of MetaboMiner's performance as well as its potential strengths and limitations.

### The effects of different types of spectral noise on compound identification

**Figure 5:**
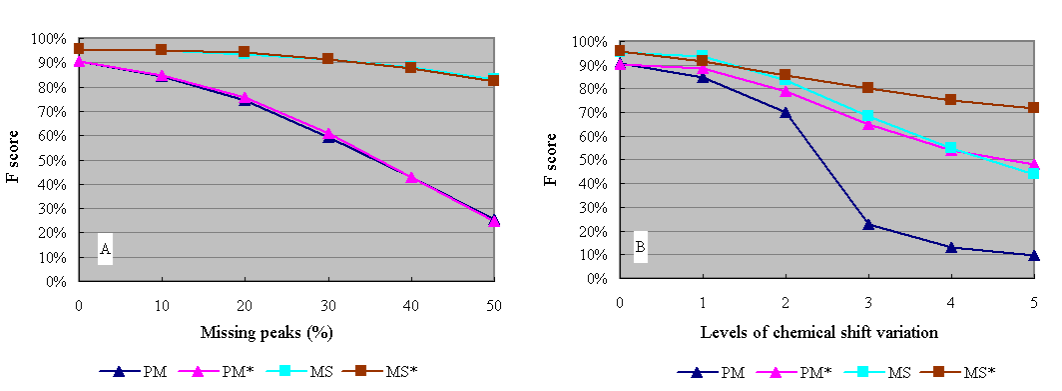
**Comparative performance of different search strategies**. Synthetic mixture query spectra were generated by pooling the peaks of 50 randomly selected compounds from MetaboMiner's reference spectral library. Different levels of spectral noise were added to these peaks and then compounds were identified with (*) and without using the adaptive threshold method. The Figure 5A, the query peaks were deleted at random with 0%, 10%, 20%, 30%, 40% and 50% probabilities; Figure 5B, the query peaks were subject to five levels of random chemical shift variations (± 0.01, ± 0.02, ± 0.03, ± 0.04, ± 0.05 ppm for each ^1^H chemical shift, and ± 0.05, ± 0.10, ± 0.15, ± 0.20, ± 0.25 ppm for each ^13^C chemical shift). The F scores were averaged over 50 iterations. (Abbreviations: PM, percentage match method; MS, minimal signature method).

The performance of the minimal signature method and the adaptive threshold method were evaluated under two common types of spectral noise – missing peaks and "drifting" peaks (i.e. peaks that have drifted from their canonical positions due to temperature, pH or solvent effects). The missing peaks were simulated by deleting peaks of each compound at random with 0%, 10%, 20%, 30%, 40%, 50% probabilities. The chemical shift drift effects were simulated by adding random values of ± 0.01, ± 0.02, ± 0.03, ± 0.04, ± 0.05 ppm for each ^1^H chemical shift, and ± 0.05, ± 0.10, ± 0.15, ± 0.20, ± 0.25 ppm for each ^13^C chemical shift. The spectra of each synthetic query mixture were generated by first pooling the peaks from 50 compounds that were randomly selected from the MetaboMiner reference spectral library (162 compounds). After introducing this artificial spectral noise, the query mixtures were searched against the reference spectral library with and without using the adaptive threshold method. Two compound identification strategies were compared – the minimal signature method (MS) and the percentage match method (PM) with 75% as the cut-off value. The F-measure was used for performance evaluation, where *F = 2 × (precision × recall)/(precision + recall) *where recall is the proportion of true positives in the returned result (*recall = TP/(TP+FN)*) and precision is a measure of the percentage of positive or correct results (*precision = TP/(TP+FP)*). The values were obtained as the averages of TOCSY and HSQC search results over 50 iterations. Figure 5A summarizes MetaboMiner's performance using data with different fractions of missing peaks. Figure 5B shows the results using data with increasing chemical shift drift effects.

### The effects of different spectral data types on compound identification

We further investigated the usefulness of different NMR data types for compound identification based on our concept of a minimal spectral signature. Four NMR data types were compared – 1D ^1^H, 1D ^13^C, ^1^H TOCSY, and ^1^H-^13^C HSQC spectra. For this particular evaluation, reference 1D ^1^H and 1D ^13^C spectra were obtained from the corresponding ^1^H and ^13^C chemical shifts of MetaboMiner's HSQC spectral library. For a small number of compounds, these artificial 1D spectra lacked some of the expected ^1^H or ^13^C signals that might be seen in a real 1D NMR spectrum, but their absence also helped to simulate the fact that some peaks in 1D NMR spectra are broadened or washed out due to signal overlap or solvent suppression.

**Figure 6:**
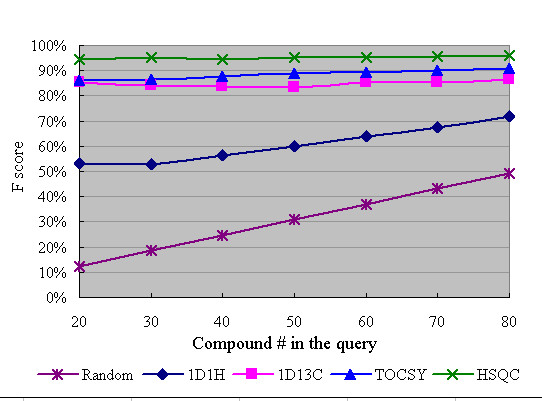
**Evaluation of MetaboMiner using simulated datasets**. Synthetic mixture query spectra were generated by pooling peaks from 20, 30, 40, 50, 60, 70, and 80 compounds randomly selected from MetaboMiner's spectral library. Spectral noise was introduced via random (10%) peak deletion and random chemical shift changes within ± 0.01 ppm for each ^1^H chemical shift, and within ± 0.05 ppm for each ^13^C chemical shift. Compound identification was based on minimal signatures using the adaptive threshold method. The F-measures were averaged over 50 iterations.

Synthetic 2D NMR spectra (query spectra) representing different biofluids of increasing molecular complexity were generated by pooling peaks of 20, 30, 40, 50, 60, 70, and 80 compounds randomly selected from MetaboMiner's reference spectral library. To further simulate noise or pH/salt effects, 10% of the peaks from the query spectra were deleted at random, followed by the introduction of random chemical shift changes (± 0.01 ppm for ^1^H and ± 0.05 ppm for ^13^C) to the remaining peaks. The resulting peaks were subsequently searched against MetaboMiner's reference spectral library using the adaptive threshold method. The F measures were averaged over 50 iterations. The result is summarized in Figure 6.

### Compound identification using experimental spectra

Twelve 2D NMR experiments (six TOCSY and six HSQC) were collected under different pH conditions using three synthetic mixtures and a plasma sample. The three synthetic mixtures were composed of 27, 21, and 24 common metabolites, respectively, with concentrations ranging from 40 to 60 mM. The plasma sample contained 35 identifiable metabolites (ranging in concentration from 0.1 to 10 mM) as determined by independent profiling of its 1D ^1^H NMR spectra by several experienced individuals using Chenomx's NMR Suite software [12]. These results were further confirmed by spiking/doping authentic standards into the plasma sample and by GC-MS analysis. The plasma sample was prepared by filtering the sample through a 3 kDa filter (to remove proteins), then lyophilizing and finally dissolving the remaining solids in distilled water to its 1/5 original volume. Deuterium oxide (D_2_O) was added to make a final concentration of 90% H_2_O and 10% D_2_O. All spectra were acquired at 25°C. Six spectra were collected on a Varian INOVA 800 MHz spectrometer equipped with a 5 mm triple axis gradient cryoprobe. The other six spectra were collected on a Varian INOVA 500 MHz spectrometer with a 5 mm triple-resonance z-gradient probe. The TOCSY experiments were performed using the wgtocsy pulse sequence, and the HSQC experiments were performed using the gChsqc pulse sequence, both provided by Varian's BioPack. For the TOCSY experiments, the spectral width was set to 11990 Hz and a mixing time of 50 milliseconds. Sixteen transients were collected for each of 128 t_1 _intervals using an acquisition time of 0.085 seconds and a relaxation delay of 2.0 seconds. The total acqisition time for the TOCSY spectra was 2.5 hours For the ^13^C-HSQC experiments, the spectral widths of the proton and carbon dimensions were 11990 Hz and 28160 Hz respectively. Sixty four transients were acquired for each t_1 _interval using an acquisition time of 0.085 seconds and a relaxation delay of 1.0 seconds. The spectra were collected with 2048*256 complex points for the ^1^H and ^13^C dimensions respectively. The total spectral acquisition time for the HSQC spectra was 5 hours. Sample TOCSY and HSQC spectra are available [see Additional File 1].

The raw NMR spectra were first processed using NMRPipe [27] and the peaks were subsequently picked using Sparky's [28] automatic peak picking program. The resulting "raw" peak lists were copied and pasted to the processing view of MetaboMiner. Both peak processing and compound identification were performed using MetaboMiner's default parameter sets. The reference library used for the synthetic mixtures was the biofluid (common) library. For plasma data, the plasma (common) library was used. To assess the degradation in performance assuming no prior knowledge of the sample source (urine, plasma, cell extract or generic biofluid) the complete spectral reference library (223 compounds for TOCSY, 502 compounds for HSQC) was also used to identify compounds. To assess the performance of the web-servers that support 2D NMR mixture analysis – the HMDB [23], the MMCD [25], the BMRB [24], and the SpinAssign [29] of PRIMe  – the same set of peak lists were submitted. For PRIMe, the default search parameters were used. For other web services, the search threshold for ^1^H was set to 0.03 ppm and 0.10 ppm for ^13^C. The results are summarized in Tables 1 and 2.

**Table 1:** Performance evaluation using HSQC data collected at pH ~7.2.

**Method**	**Sample**	**# Cmpds**	**#Correctly Identified (TP)**	**FN**	**FP**	**Precision (%)**	**Recall (%)**	**F score**
MetaboMiner-sp	A	27	21	6	6	77.8	77.8	77.8
MetaboMiner-all	A	27	15	12	8	65.2	55.6	60.0
HMDB	A	27	8	19	19	29.7	29.7	29.7
MMCD	A	27	8	19	6	57.1	29.7	39.1
BMRB	A	27	6	21	21	22.2	22.2	22.2
PRIMe	A	27	14	13	6	70.0	51.9	59.6
MetaboMiner-sp	B	21	16	5	5	76.2	76.2	76.2
MetaboMiner-all	B	21	9	12	0	100	42.9	60.0
HMDB	B	21	1	20	20	4.8	4.8	4.8
MMCD	B	21	0	21	0	0	0	0
BMRB	B	21	1	20	20	4.8	4.8	4.8
PRIMe	B	21	6	15	4	60.0	28.6	38.7
MetaboMiner-sp	C	24	22	2	2	91.7	91.7	91.7
MetaboMiner-all	C	24	16	8	3	84.2	66.7	74.4
HMDB	C	24	9	15	15	37.5	37.5	37.5
MMCD	C	24	2	22	0	100	7.4	13.8
BMRB	C	24	3	21	21	12.5	12.5	12.5
PRIMe	C	24	8	16	5	61.5	33.3	43.2
MetaboMiner-sp	D	35	29	6	6	82.9	82.9	82.9
MetaboMiner-all	D	35	16	19	7	69.6	45.7	55.2
HMDB	D	35	9	26	26	25.7	25.7	25.7
MMCD	D	35	4	31	3	57.1	11.4	19.0
BMRB	D	35	7	28	28	20.0	20.0	20.0
PRIMe	D	35	14	21	5	73.7	40.0	51.8

Samples A, B, and C are synthetic cocktail mixtures and sample D is a plasma sample.

(Annotation: MetaboMiner-sp = searched using the biofluid-specific library; MetaboMiner-all = searched using the entire spectral library; TP = true positives; FN = false negatives; FP = false positives).

**Table 2:** Performance evaluation using TOCSY data collected at pH ~7.2.

**Method**	**Sample**	**# Cmpds**	**# Correctly Identified (TP)**	**FN**	**FP**	**Precision (%)**	**Recall (%)**	**F score**
MetaboMiner-sp	A	27	23	4	4	85.2	85.2	85.2
MetaboMiner-all	A	27	21	6	6	77.8	77.8	77.8
HMDB	A	27	2	25	25	7.4	7.4	7.4
MMCD	A	27	1	26	26	3.7	3.7	3.7
MetaboMiner-sp	B	21	16	5	5	76.2	76.2	76.2
MetaboMiner-all	B	21	12	9	2	85.7	57.1	68.5
HMDB	B	21	2	19	19	9.5	9.5	9.5
MMCD	B	21	2	19	19	9.5	9.5	9.5
MetaboMiner-sp	C	24	17	7	7	70.8	70.8	70.8
MetaboMiner-all	C	24	15	9	8	65.2	62.5	63.8
HMDB	C	24	4	20	20	16.7	16.7	16.7
MMCD	C	24	2	22	22	8.3	8.3	8.3
MetaboMiner-sp	D	35	30	5	5	85.7	85.7	85.7
MetaboMiner-all	D	35	23	12	12	65.7	65.7	65.7
HMDB	D	35	3	32	32	8.6	8.6	8.6
MMCD	D	35	2	33	33	5.7	5.7	5.7

Samples A, B, and C are synthetic cocktail mixtures and sample D is a plasma sample.

(Annotation: MetaboMiner-sp = searched using the biofluid-specific library; MetaboMiner-all = searched using the entire spectral library; TP = true positives; FN = false negatives; FP = false positives).

## Results

Using synthetic query spectra constructed as described previously, the performance characteristics of MetaboMiner were first assessed under different levels of spectral noise. Secondly, the utility of different NMR data types were also investigated for our approach of compound identification. Finally, we evaluated MetaboMiner's performance using a total of 12 real NMR spectra collected from defined compound mixtures and a plasma sample of known composition.

After creating the spectral reference libraries and calculating the uniqueness values for each peak, we first investigated the performance of the minimal signature (MS) method versus the adaptive threshold method under different types of spectral noise. As an additional comparison, the percentage match (PM) method was also included. As shown in Figure 5, the MS method performed consistently better than the PM method when missing peak and chemical shift variations are present. The adaptive threshold method appears to be most effective for data exhibiting large chemical shift variations. When chemical shift variation is negligible, as in the test with missing peak data, this method performs exactly the same as the fixed threshold method.

The utility of different NMR data types was also investigated using synthetic spectra of increasing complexity. As illustrated in Figure 6, HSQC, TOCSY and ^13^C-based methods worked very well over the full range of compound mixtures. The number of compounds in the query mixture has very little effect on their overall performance. The slight increases in F scores with increasing spectral complexity are not statistically significant. In general, MetaboMiner's performance for compound identification was best using the HSQC dataset. It is also apparent that the minimal signature method favours compound identification with HSQC spectra as these spectra tend to have more unique peaks than other types of NMR spectra. Also evident from Figure 6 is the fact that MetaboMiner's performance using 1D ^1^H data, alone, is the poorest. In contrast to the ^1^H spectra (both 1D and 2D), it is quite clear that ^13^C chemical shifts (even in 1D spectra) can provide sufficient information for robust compound identification. This is mainly due to the much wider chemical shift dispersion (0~200 ppm) seen in ^13^C spectra compared to ^1^H spectra. Most ^13^C chemical shifts remain unique even in mixtures of 162 compounds.

While ^13^C spectra (1D and 2D) provide excellent data sets for compound identification we found that by focusing on off-diagonal peaks originating from the coupling between pairs of protons, the utility of TOCSY spectra could be greatly improved. As indicated in Figure 6, MetaboMiner's metabolite identification performance based on TOCSY spectra was better than that based on 1D ^13^C spectra and was only slightly outperformed by ^13^C HSQC data. These results underscore the utility of using 2D spectra in NMR-based compound identification of complex (>20 compounds) mixtures.

We also assessed MetaboMiner's performance on its own and against several other web services using eight experimental NMR spectra collected from four different mixtures of known composition. As indicated in Tables 1 and 2, the best performance for MetaboMiner was obtained when a biofluid-specific reference library was used to analyze TOCSY or HSQC data. On average, MetaboMiner was able to correctly identify (recall, precision and F-measure) an average of 81% of the compounds from both TOCSY and HSQC data. When the entire spectral library (223 TOCSY, 502 HSQC) was used, the performance (F-measure) decreased by an average of 15%. Among the four web services evaluated using the same data (Table 1), the SpinAssign program performs the best (F-measure = 49%) but this is still about 30% worse than MetaboMiner when it uses a biofluid-specific library and 15% worse than MetaboMiner when it uses its entire spectral library. Overall, the other web servers did not perform particularly well with average F-measures of 15–25% for HSQC data and 6–12% for TOCSY data. Both the HMDB and MMCD servers performed better when analyzing HSQC data than TOCSY data. The performance for all web servers was essentially the same regardless of whether "clean" peak lists (no noise peaks) or "raw" peak lists were used as input. Note that TOCSY mixture analysis is not currently supported by either PRIMe or BMRB.

In an effort to understand the influence of pH on the efficacy of compound identification by 2D NMR, we also collected TOCSY and HSQC data at pH 4.2 and pH 8.8. This is approximately 3 pH units below (and 1.5 pH units above) the pH at which the spectral library standards were collected. As seen in Table 3, a significant pH change in the sample (relative to the pH of the spectral libraries) can negatively impact the performance of compound identification. For instance, at pH 4.2, the F-measure drops by more than 20% for the HSQC spectra.

**Table 3:** Performance evaluation of MetaboMiner under different pH conditions.

**Sample**	**# Cmpds**	**NMR Exp.**	**pH**	**# Correctly Identified (TP)**	**FN**	**FP**	**Precision (%)**	**Recall (%)**	**F score**
A	27	HSQC	4.2	15	12	12	55.6	55.6	55.6
A	27	HSQC	7.2	21	6	6	77.8	77.8	77.8
A	27	TOCSY	4.2	21	6	6	77.8	77.8	77.8
A	27	TOCSY	7.2	23	4	4	85.2	85.2	85.2
D	35	HSQC	7.3	29	6	6	82.9	82.9	82.9
D	35	HSQC	8.8	24	11	4	85.7	68.6	76.2
D	35	TOCSY	7.3	30	5	5	85.7	85.7	85.7
D	35	TOCSY	8.8	25	10	6	80.6	71.4	75.7

Tests were performed using biofluid-specific library.

## Discussion

In this paper, we described the development and assessment of a software tool (MetaboMiner) to facilitate compound identification from 2D NMR spectra of biofluids or small molecule mixtures. We first created a series of "clean" spectral reference libraries based on publicly available spectral databases. Secondly we developed and tested several algorithms to facilitate robust and automatic peak processing, peak matching and compound identification. Finally, we integrated these resources into an easy-to-use application and evaluated its performance using a variety of synthetic and real spectra.

MetaboMiner's spectral reference library covers most NMR-detectable metabolites present in human biofluids [23]. To improve the reliability of the compound identification, the larger spectral library was further partitioned into three smaller libraries based on the composition of different biofluids (CSF, plasma and urine). In addition, we also created "common" libraries for each type of biofluid that contain the most common or abundant metabolites found in these biofluids (as ascertained from previous experience and from data contained in the HMDB). We found that the creation of biofluid-specific libraries significantly improved compound identification by reducing the spectral search space. As indicated in Tables 1 and 2, a 15% reduction in MetaboMiner's performance occurred if the entire compound library was used instead of the biofluid-specific "common" library. For researchers who wish to study other types of biofluids, MetaboMiner provides intuitive interfaces that allow users to easily expand and customize their spectral reference libraries.

### Performance Assessment

The performance of MetaboMiner was evaluated using a variety of synthetic and experimental datasets. In all cases, our strategies for peak matching and compound identification showed robust performance under various noise (real and synthetic) conditions. Using synthetic data, the best compound identification performance was ~90% (F-measure) under moderate noise levels as indicated by Figure 6. Further inspection of the compound identification lists showed that the most common problem was the identification of false positives. In particular, for certain compounds it is inherently difficult to uniquely identify them by NMR based on their matched peaks. For example, the TOCSY peaks of citrate and serine cluster very tightly around the diagonal. They also overlap with peaks of other more abundant compound species. As a result, they are often misidentified. Another source of false positives comes from the existence of structurally similar compounds such as asparagine/aspartate, inosine/adenosine, or creatine/creatinine, which have nearly identical NMR spectral features.

When we assessed the performance of MetaboMiner using experimentally collected data, the performance was reduced by ~10% for both TOCSY and HSQC data. Close examination of the lists of identified compounds as well as the spectra used in the evaluation indicated two sources of problems (see Additional File 2). The first relates to the fact that several compounds known to be in the mixtures failed to be identified (false negatives). Manual inspection of the actual spectra (TOCSY or HSQC) indicated that in every case, no peaks or very weak peaks were visible for these compounds. These compounds were obviously below the detection threshold of the instrument. The second problem was the existence of several false positives. Again, manual inspection of the spectra showed that the false positives were mainly caused by spectral artefacts. Real spectra [see Additional File 3] can contain a significant amount of spectral noise. In the case of TOCSY spectra, most of the automatically picked peaks (>60%) are from these artefacts. Obviously if cleaner spectra could be collected or if more manual intervention was used to eliminate some of the spectral artefacts prior to submitting the data to MetaboMiner, a better performance could be achieved. In addition, we also observed that some compounds are exquisitely sensitive to small pH variations such as lactate, uracil and histidine.

### Peak uniqueness and the challenges in automated compound identification

There are three major challenges facing automatic compound identification for NMR-based metabolomics. The most common and perhaps the most vexing is the so-called spectral overlap problem. The spectral complexity inherent in many biofluids can lead to a large number of peaks confined to a relatively narrow chemical shift range (~10 ppm for ^1^H spectra). With the increased availability of high-field NMR spectrometers, the problem of spectral overlap is diminished somewhat for simple biofluids such as CSF. However, for complex mixtures like plasma, urine, or tissue extracts, the problem is still quite severe. The second challenge in automated compound identification is the handling of chemical shift changes induced by the variation of pH, temperature, ionic strength, *etc*. This effect combined with the first issue makes it difficult to perform automated compound identification based solely on chemical shifts. The third challenge in automated compound identification is the low signal-to-noise (S/N) ratio for the NMR resonances of low abundance compound species. Consequently, the wide range of metabolite concentrations found in many biofluids poses a serious problem for most automatic peak-picking programs. In particular, many low intensities peaks are likely to be missed by most peak picking programs.

To address these challenges we introduced the notion of "uniqueness" in the reference library in order to deal with the problem of missing peaks and chemical shift variations. The uniqueness values were calculated for every peak based on their relative distances to each other in a given reference library. Although a finer scale may perform better for more complex mixtures, we found that the use of five levels of uniqueness works well for most situations. In MetaboMiner, both peak matching and compound identification rely heavily on these uniqueness values. During peak matching, an adaptive threshold method was used to adjust the current search threshold to its maximum uniqueness scope. This approach significantly improves MetaboMiner's performance when chemical shift variations are nontrivial. By using an adaptive threshold method we found the effect of chemical shift changes or chemical shift drift could be tolerated to a greater extent. These uniqueness values also allow us to derive minimal signatures based on a relatively small portion of unique peaks. This approach turned out to be both robust and flexible since it did not require "stable" spectral patterns. In contrast, the common percentage match (PM) method weights each peak equally and uses a fixed threshold for compound identification. This approach suffers greatly if a nontrivial proportion of peaks are not matched. Note that the minimal signature method in MetaboMiner is complemented by authenticity checks with due consideration of the total matched patterns.

It should be noted that the performance of MetaboMiner strongly depends on the quality of the upstream spectral collection and processing work. As a general rule, for optimal performance, the NMR experiments on biofluid mixtures should be carried out under the same (or at least similar) conditions as the conditions used to collect the spectra for the reference library (i.e. neutral pH). The automatic peak-picking process should be closely monitored and an iterative approach is recommended in order to pick up most signals while avoiding obvious spectral noise. In our testing process, a typical TOCSY spectrum usually generated 2,000~3,000 peaks with a high proportion of noise peaks. We found that these noisy signals were handled quite efficiently by MetaboMiner. As a general rule, we would suggest that users employ a low threshold during the peak picking stage for TOCSY spectra. For HSQC spectra, because of the difficulty associated with detecting and removing noisy signals, we would suggest more manual intervention during the peak picking process.

### Comparison to other spectral analysis software tools

There are several commercial software tools available for analysing complex metabolite mixtures using NMR. Chenomx *Inc*. has developed a commercial bioprofiling software package called the Chenomx NMR Suite that allows semi-automated identification of compounds from 1D ^1^H NMR spectra. The Chenomx software package provides an excellent interface for compound identification and quantification via a manual peak-fitting process using a spectra reference library containing 260 compounds [12]. However, the requirement for manual fitting and analysis leaves the process open to inconsistent interpretation or inconsistent assignment by different individuals. Furthermore, the analysis can take upwards of one hour per sample and the software is relatively expensive. Bruker's AMIX (Bruker BioSpin) software is another powerful tool that offers support for compound identification and quantification for 1D and 2D NMR as well as LC-MS spectra. It used a method called AutoDROP to facilitate compound identification and structure verification [30]. The key idea is the systematic decomposition of reference spectra into spectral patterns of molecular fragments. Compound identification is based on recognition of such patterns in the target spectra. However, similar limitations pertaining to cost, the reliance on manual analysis, processing time and inconsistent interpretation appear to apply to AMIX as well.

In addition to these commercial packages there is at least one other non-commercial system that has been described. Xi *et. al *[24] developed a statistical and chemical model for automatically identifying compounds in mixtures using 2D COSY spectra. Like MetaboMiner, the Chenomx NMR Suite and AMIX, Xi *et al*'s method uses a library of pre-collected NMR spectra to assist with compound identification. These authors reported experimental results using spectra collected from abalone muscle and digestive gland extracts. Their method was able to identify 12 out of 15 amino acids and 6 out of 9 amino acids as determined with Chenomx's NMR Suite. However, it appears that this system has a spectral library of only 19 COSY spectra, so it is very limited in terms of practical applications.

In addition to these stand-alone, graphically based software packages, several metabolomics database websites, including the HMDB [23], MMCD [25], BMRB [24], and PRIMe now allow direct querying of their databases using peak lists obtained from compound mixtures. However, as web servers they are somewhat limited in their graphic capabilities and user-interface interactions. In particular, most of these sites typically return long lists of potentially matched compounds without a graphic display of the matched spectra to help users make their decisions. Further, most of the servers appear to be designed to handle single compound or simple mixture queries and are not optimized for compound identification from complex biofluid mixtures. This is particularly evident from the results shown in Tables 1 and 2. The modest performance for the MMCD and BMRB servers with regard to their handling of HSQC spectra may be partially due to the pH and solvent differences between the query spectra and their internal reference spectra (pH 7.0–7.2 in H_2_O vs. pH 7.4 in D_2_O), however these pH differences are relatively small and should not have affected the identification of most compounds. Furthermore, the fact that the same level of performance was seen with TOCSY queries where MetaboMiner, BMRB and MMCD used the same set of reference spectra and had the same pH mistmatch suggests that the performance differences are likely related to algorithmic robustness. Likewise the relatively good performance of the PRIMe server is likely due to the fact that it only contains spectra for 80 common compounds. This significantly reduces the number of false positives and somewhat inflates its overall performance.

Overall, MetaboMiner combines many of the useful interactive graphic features and high levels of performance of the stand-alone commercial packages such as Bruker's AMIX and Chenomx's NMR Suite with the relatively simple automation or semi-automation seen with NMR-based metabolomics web servers. Key to MetaboMiner's success are its large and carefully constructed spectral libraries, its robust spectral filtering and peak matching routines, and its use of biofluid-specific spectral libraries to rationally limit the spectral search space. Given the extensive testing and the availability of many built-in tools for spectral manipulation, viewing and annotation we believe MetaboMiner is well designed and ready for practical metabolomics applications.

## Limitations

MetaboMiner is not without its limitations. In particular, MetaboMiner does not support compound quantification. Efforts are underway to add this functionality to the program but it appears that quantification via 2D NMR spectra is intrinsically more difficult and less reliable than with 1D NMR spectra. Lewis et al. [31] recently described a quantification method for HSQC spectra using standard curves calibrated to unique or selected peaks of a given compound. Quantification by TOCSY still remains difficult because the magnetization transfer functions for complex spin systems can differ greatly between metabolites. This can lead to widely different peak intensities. Another limitation to MetaboMiner (which is also shared by other NMR analysis programs such as Chenomx's NMR Suite and Bruker's AMIX) is the fact that MetaboMiner's performance is highly dependent on the spectral quality and spectral pre-processing of the query (i.e. experimental) spectra. High signal-to-noise, good phasing, minimal baseline distortion and the elimination of spectral artefacts will always improve the performance. However, some biofluid samples may be refractory to good spectral processing or some users may lack sufficient experience/skill to properly process their spectra. Under these circumstances, MetaboMiner's results may prove to be unreliable or non-reproducible. Overall, the performance for MetaboMiner (and even for human experts) degrades as the signal-to-noise ratio for the peaks of any given compound drops below 3:1. The lower limit of detection on our 500 MHz instrument for TOCSY data was about 50 mM and about 100 mM for HSQC data. A third limitation to MetaboMiner is its limited sensitivity. In particular, MetaboMiner's exclusive reliance on 2D NMR spectra generally reduces its sensitivity limit by a factor of ~10 over what might be detected via 1D spectrum. Obviously the use of more concentrated samples, longer collection times or isotopic labelled samples can overcome these problems, but the trade-off between time, cost and convenience may not always be in MetaboMiner's favour. It is worth noting that Methods such as ASAP HMQC [32] now allow very rapid acquisition of 2D NMR data. This may make the issues of sensitivity and time much less important, particularly for 2D heteronuclear experiments. A fourth limitation is that fact that MetaboMiner's spectral libraries (esp. the TOCSY library) are still missing a number of important compounds. Efforts are underway to expand the TOCSY library over the coming months and the MetaboMiner website will provide periodic reference spectral updates. Alternately, users are invited (and encouraged) to add their own spectra to MetaboMiner's reference libraries. Finally, unlike Chenomx's NMR Suite and Bruker's AMIX, MetaboMiner's spectral libraries do not cover a broad range of pH values. As a result, MetaboMiner is largely restricted to analyzing spectra from biofluids or metabolite mixtures that are titrated to pH 7.0 +/- 0.5.

## Conclusion

In this paper we have demonstrated that by utilizing the extra information found in 2D NMR spectra as well as prior knowledge about the composition of the biofluid itself, it is possible to semi-automatically identify a significant number of compounds in complex aqueous mixtures (both defined mixtures and biofluids) with excellent (>80%) accuracy. In particular, the quality and degree of metabolite identification achieved by MetaboMiner certainly matches that of what a skilled NMR spectroscopist could do – but in significantly less time. Overall, we have shown that by using a comprehensive reference library coupled with robust algorithms for peak processing, peak matching and compound identification, the process of metabolite identification from 2D NMR spectra can be greatly simplified.

## Availability and requirements

Project name: MetaboMiner

Project home page: 

Operating system: Platform independent

Programming language: Java

Other requirements: Java 1.5 or higher

License: GPL

## Abbreviations

CSF: cerebrospinal fluid; DSS: 2, 2-Dimethyl-2-silapentane-5-sulfonic acid; GUI: graphic user interface; HPLC: high performance liquid chromatography; HSQC: heteronuclear single quantum correlation spectroscopy; MS: mass spectrometry; NMR: nuclear magnetic resonance; PNG: portable network graphics; ppm: parts per million; TOCSY: total correlation spectroscopy; TSP: trimethylsilyl-2, 2, 3, 3-tetradeuteropropionic acid; UPLC: ultra-high performance liquid chromatography; GC-MS: gas chromatography mass spectrometry; LC-MS: liquid chromatography mass spectrometry.

## Authors' contributions

JX carried out the software design, implementation, and drafted the manuscript. TB participated in generating 2D experimental data. PT participated in the implementation of the user interfaces and evaluated the software. DSW conceived of the study, participated in the creation of spectral library and implementation of search algorithm, edited the manuscript and reviewed the software. All authors read and approved the manuscript.

## Supplementary Material

Additional file 1 **Supplemental figure 1** Click here for file

Additional file 2 **Synthetic mixture information** Click here for file

Additional file 3 **Supplemental figure 2** Click here for file
