# Supplementary figures and images for: MetaboMiner – semi-automated identification of metabolites from 2D NMR spectra of complex biofluids
Source: BMC Bioinformatics. 2008 Nov 28;9:507. doi: 10.1186/1471-2105-9-507 (PMC2612014; doi:10.1186/1471-2105-9-507)

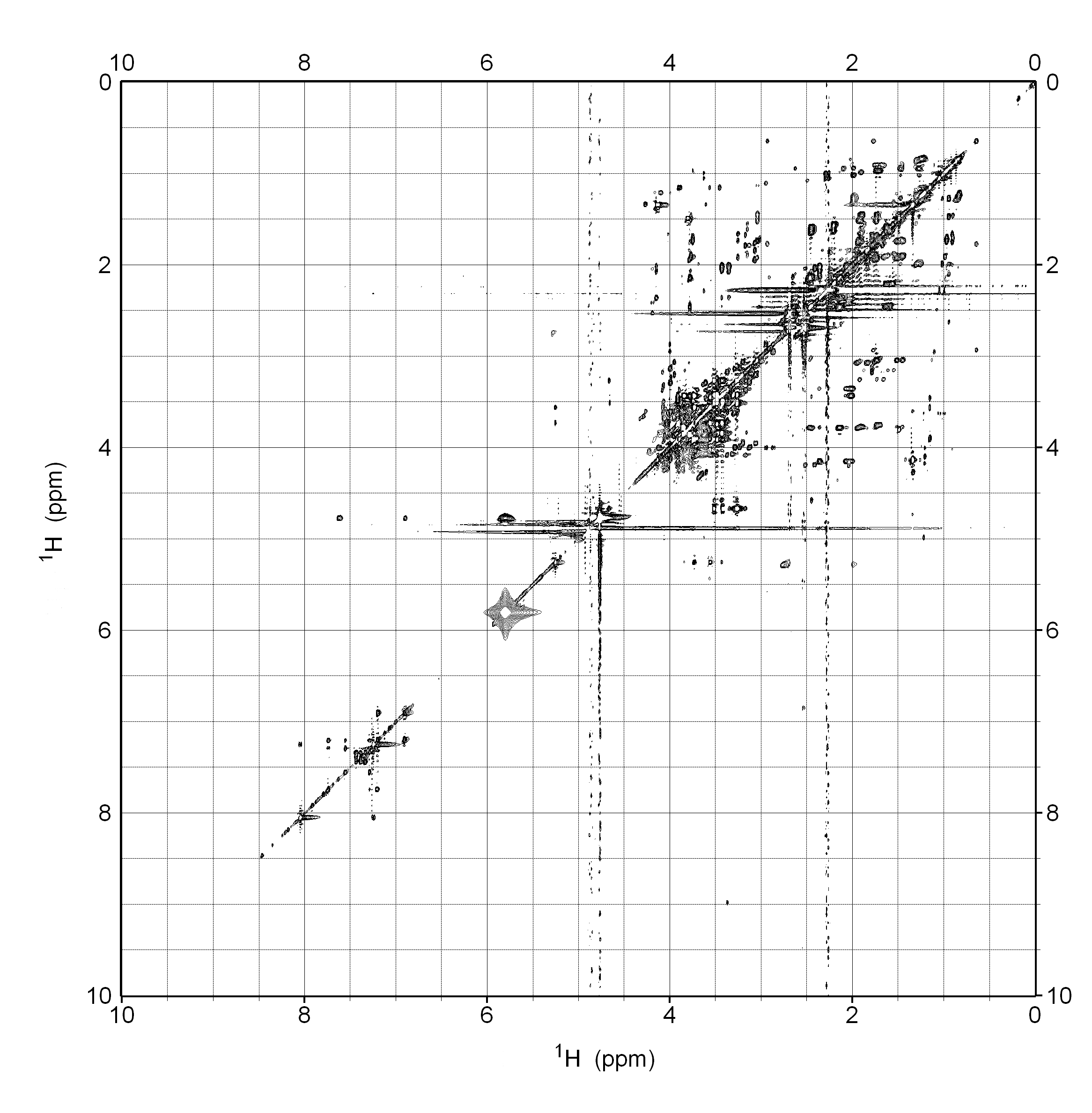

Supplement: Additional file 1 — Supplemental figure 1 [file 1471-2105-9-507-S1.png]

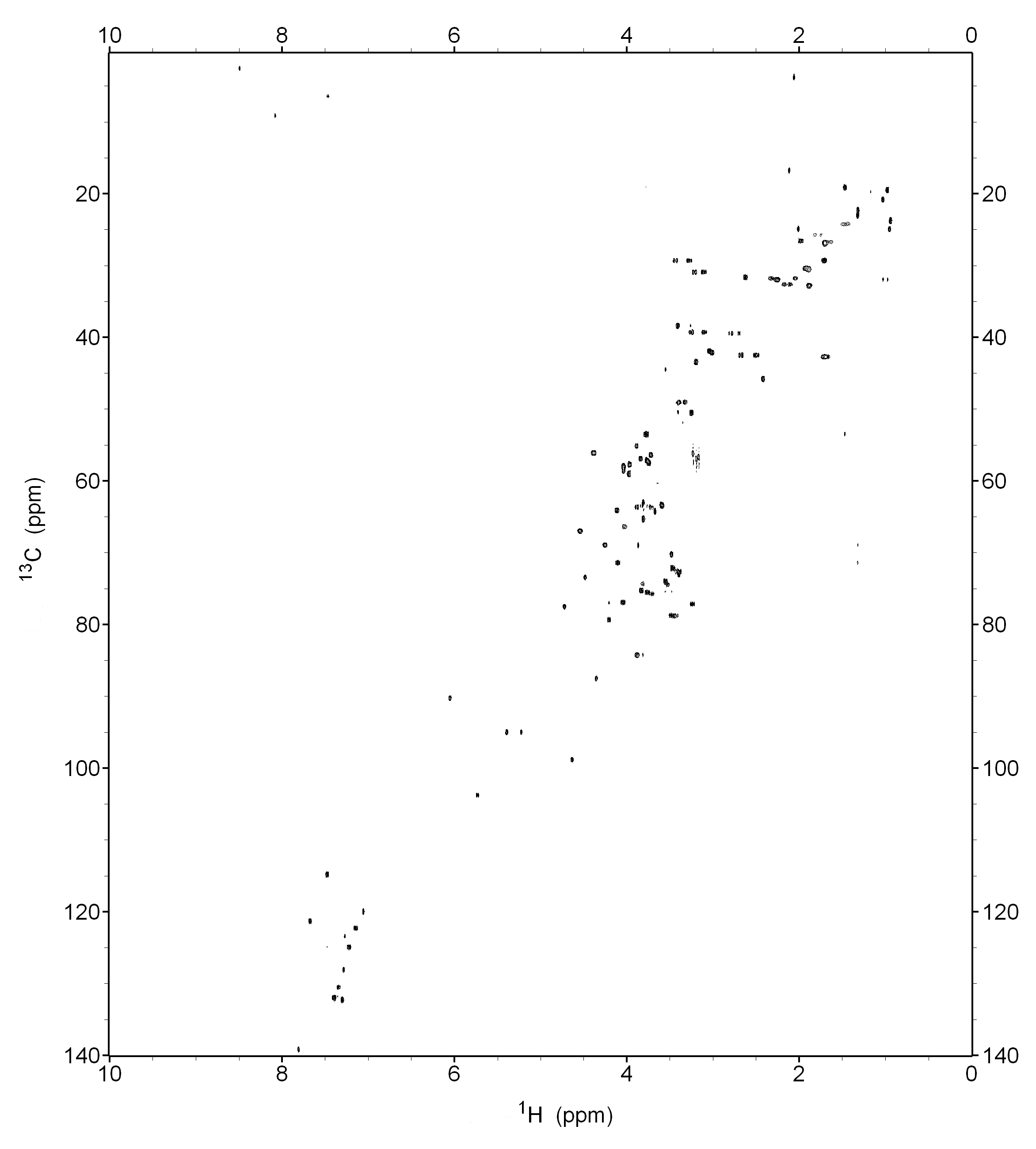

Supplement: Additional file 3 — Supplemental figure 2 [file 1471-2105-9-507-S3.png]
